# Challenging Anatomies for TAVR—Bicuspid and Beyond

**DOI:** 10.3389/fcvm.2021.654554

**Published:** 2021-04-13

**Authors:** Mohammed Saad, Hatim Seoudy, Derk Frank

**Affiliations:** ^1^Department of Internal Medicine III, Cardiology and Angiology, University Hospital Schleswig-Holstein, Kiel, Germany; ^2^DZHK (German Centre for Cardiovascular Research), Partner Site Hamburg/Kiel/Lübeck, Kiel, Germany

**Keywords:** anatomy, aortic stenosis, bicuspid aortic valve, transcatheter aortic valve implantation, transcatheter aortic valve replacement

## Abstract

Transcatheter aortic valve replacement has emerged as the standard treatment for the majority of patients with symptomatic aortic stenosis. As transcatheter aortic valve replacement expands to patients across all risk groups, optimal patient selection strategies and device implantation techniques become increasingly important. A significant number of patients referred for transcatheter aortic valve replacement present with challenging anatomies and clinical indications that had been historically considered a contraindication for transcatheter aortic valve replacement. This article aims to highlight and discuss some of the potential obstacles that are encountered in clinical practice with a particular emphasis on bicuspid aortic valve disease.

## Introduction

Since its introduction in 2002 by Cribier et al., transcatheter aortic valve replacement (TAVR) has rapidly evolved into an essential treatment option for patients with symptomatic aortic stenosis (AS) (1–3). While TAVR was initially limited to patients at high or prohibitive surgical risk, there is growing evidence supporting the use of TAVR in intermediate and selected low-risk patients (4–7). As a result, TAVR has been fully integrated into current guidelines for the management of valvular heart disease (8, 9). Due to substantial advances in technology and technique, TAVR can now be safely performed in an expanding population with aortic valve disease. Nevertheless, a significant number of AS patients present with complex anatomical features that may hamper the successful use of TAVR.

## Challenges in Femoral Access

Transfemoral (TF) access is the most commonly used and best studied approach for TAVR. TF access is accepted as the gold standard and can be successfully achieved in the vast majority of AS patients (8, 9). A subgroup of ~10–15% of TAVR candidates, however, may not be eligible for TF TAVR due to unfavorable anatomies (10). These mainly comprise (a) small native vessel size, (b) severe peripheral artery disease, and (c) extensive vessel and aortic tortuosity. In patients with challenging anatomies for a TF approach, various techniques are available. In the absence of adequate randomized clinical trials, the optimal strategy in the context of challenging access anatomy remains largely operator-dependent. In complex cases, the heart team should evaluate the use of an alternative, non-TF access site, such as a transcarotid, transsubclavian/transaxillary, transaortic, transapical, or transcaval approach, but also reconsider surgical aortic valve replacement (SAVR).

### Small Native Vessel Size

Minimal vessel dimensions required for successful TF access largely depend on the delivery system and the size of the transcatheter heart valve (THV). Using contemporary low-profile sheath designs, the minimal vessel size may be as low as 5.0 mm (CoreValve Evolut R system when using Model ENVEOR-U, Medtronic) and 5.5 mm (14F eSheath, Edwards Lifesciences). With the integrated sheath of the novel FlexNav delivery system (FlexNav™ DS) of the Portico THV system, the insertion diameter is quite similar as that of the Evolut R system, allowing a TF access down to 5.0 mm vessels (11).

**Figure 1:**
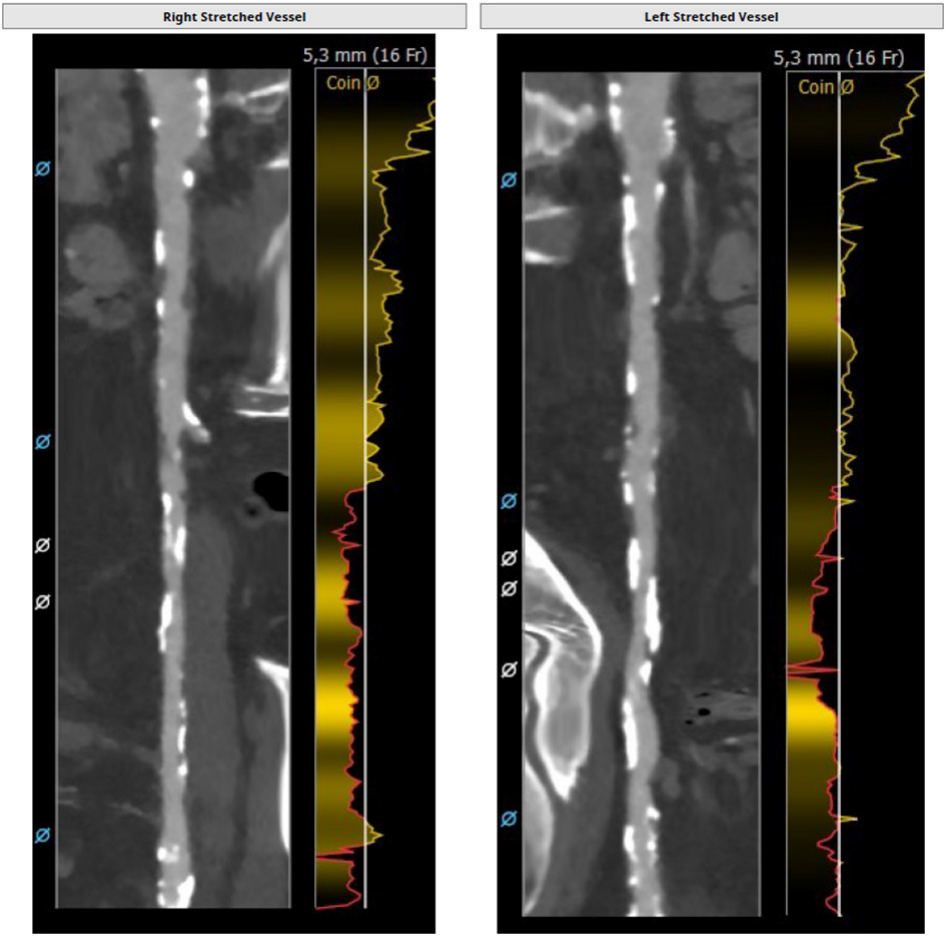
Severe peripheral artery disease with small vessel size.

Especially in patients with small vessels, preprocedural computed tomography (CT) imaging with three-dimensional reconstruction is crucial to correctly determine the luminal diameter (Figure 1). In addition, we strongly advocate the use of ultrasound guidance to identify the optimal puncture site of the femoral artery which is typically located below the inguinal ligament and ~1–2 cm above the femoral bifurcation (12). The most suitable access point may vary though and should be based on careful planning which may also include an iliac angiogram using the contralateral site in selected cases.

### Severe Peripheral Artery Disease

Peripheral artery disease (PAD) is frequently found in AS patients, as both entities share common cardiovascular risk factors such as age, smoking, hypertension, diabetes, and chronic kidney disease (13). Among high-risk AS patients, significant PAD has been reported to be prevalent in ~25% of patients undergoing TF TAVR. In addition, PAD is associated with increased mortality, bleeding complications and readmission rates after TAVR (14). In particular, circumferential calcifications may (a) interfere with adequate vessel puncture, (b) compromise sheath or device passage, and (c) increase the risk of major vascular complications such as dissection, bleeding and plaque disruption with acute limb ischemia (15) (Figure 1). In addition, anterior, posterior and especially circumferential calcification of the femoral artery reduces the efficacy of percutaneous suture-based closure devices (16). Strategies such as balloon angioplasty using the contralateral femoral artery as well as intravascular lithotripsy have been suggested to expand TF access to a subgroup of patients with significant PAD (17, 18). Alternatively, surgical femoral cut-down with or without surgical endarterectomy may be used for heavily calcified and tortuous peripheral vessels as it allows for direct visualization of the arterial access site and surgical vessel repair if needed. However, there is a lack of sufficient data to promote this approach.

### Extensive Vessel and Aortic Tortuosity

**Figure 2:**
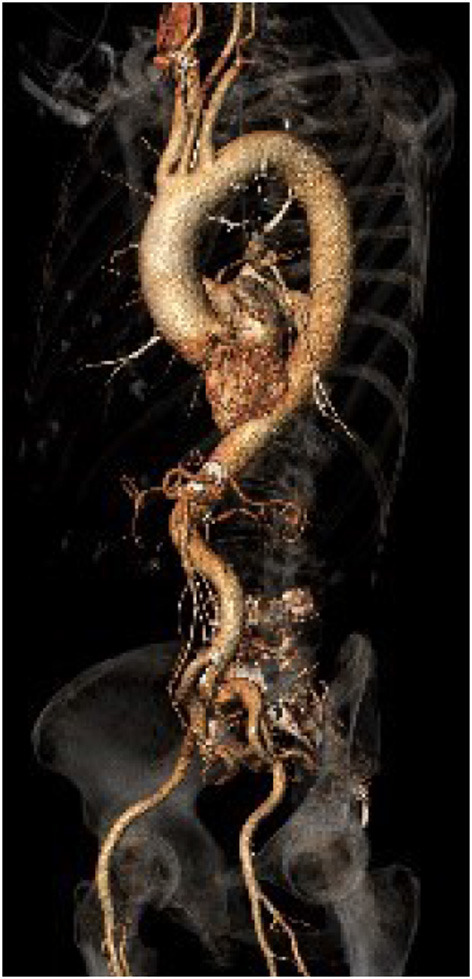
Excessive vessel tortuosity with an S-shaped aorta.

Vessel tortuosity may prevent successful TF TAVR and in extreme cases is associated with increased vascular complications such as dissection, rupture and life-threatening bleeding (19) (Figure 2). As there are no specific cut-offs for prohibitive vessel tortuosity, the optimal access strategy should be established on a case-by-case basis and remains largely operator-dependent. In many patients with circumscribed vessel tortuosity, the use of a long delivery sheath allows for a safe TF access. In case of more pronounced tortuosity of the iliofemoral arteries or the aorta (including an S-shaped aorta), an extra-stiff guidewire, such as the Lunderquist® Extra Stiff (Cook Medical), Back-up Meier™ (Boston Scientific), or Amplatz Ultra Stiff® (Cook Medical), may be employed to straighten the aorto-ilio-femoral axis. In extreme scenarios, the use of two or multiple extra-stiff guidewires, commonly referred to as the “buddy wire technique,” is a viable strategy (20). As extreme vessel tortuosity predisposes to injury to the access route, careful pre- and post-procedural assessment including CT, aortography and/or ultrasound is mandatory.

## Challenges in Aortic Anatomy

Complex aortic anatomy may significantly impede device delivery and accurate implantation of a THV. Two major challenges of aortic anatomy comprise (a) horizontal aorta and (b) concomitant aortic aneurysm.

### Horizontal Aorta

**Figure 3:**
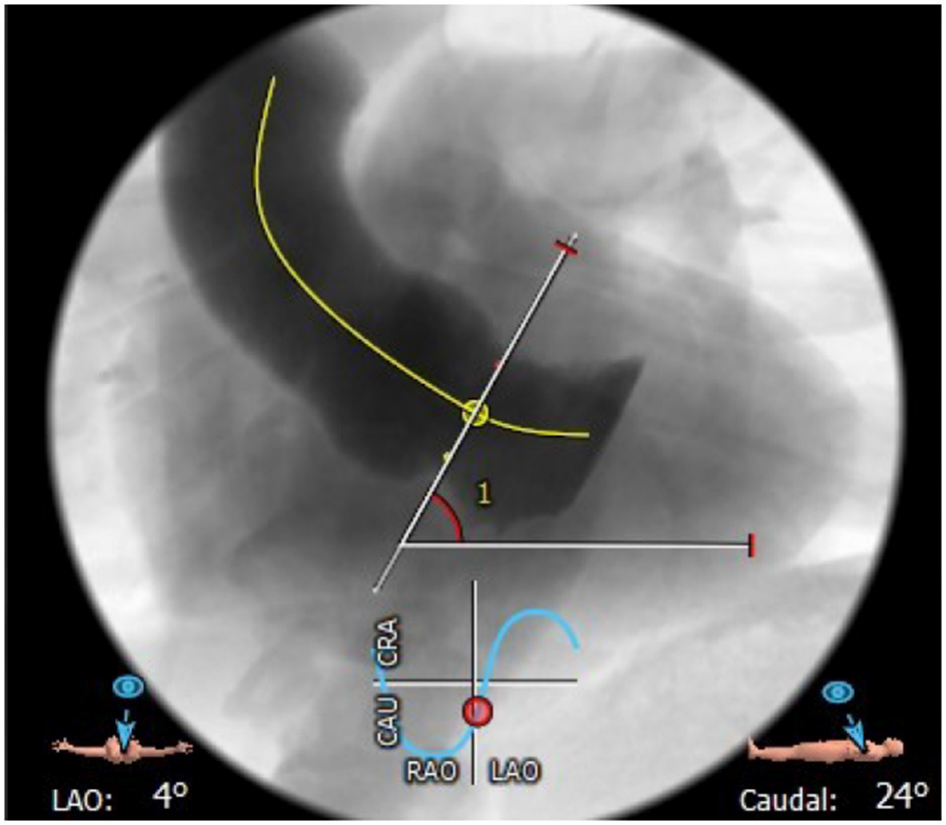
Horizontal aorta (aortic angulation 61°).

Horizontal aorta refers to an excessive aortic angulation measured in coronal projection (Figure 3). Based on data of predominantly older-generation TAVR devices, horizontal aorta was initially defined as an angulation of ≥48° between the horizontal plane and the plane of the aortic annulus (21). Using this threshold, the authors found an inverse relationship between aortic angulation and procedural success of self-expanding, but not balloon-expandable THVs. In theory, this may be explained by the longer stent frame and the non-steerable delivery catheter which may lead to suboptimal valve positioning compared to devices such as the SAPIEN 3. However, conflicting results have been published regarding the impact of a horizontal aorta on outcomes and success rates using newer-generation devices, suggesting a comparable safety profile between self-expanding and balloon-expandable prostheses (22–24). Moreover, the Acurate-NEO self-expanding THV is initially released from the aorta rather than from the LVOT, with subsequent deployment of the sub-annular portion, thus preventing a deep implant into the left ventricle; additionally, the 3 stabilization arches safely facilitate axial alignment which can be challenging in extreme horizontal aortas (25).

While in extreme forms of horizontal aorta the use of a SAPIEN 3 device is often preferred by implanting operators, this is not supported by currently available data. In the absence of randomized clinical studies and clear definitions of a horizontal aorta, the type of TAVR prosthesis therefore remains largely operator-dependent. Moreover, the optimal management of patients with extreme forms of a horizontal aorta (e.g., ≥70°) is still unclear and should be based on the local expertise of the heart team. Especially in patients with a horizontal aorta and additional anatomical features such as bicuspid aortic valve (BAV) or a complex calcification pattern, the use of a non-TF access or SAVR should be considered. In addition, future studies are necessary to evaluate the impact of increased aortic angulation on long-term valve function, durability, and the feasibility of valve-in-valve (ViV) procedures and coronary access.

### Concomitant Aortic Aneurysm

Dilatation of the ascending aorta (>40 mm) is frequently found in patients with AS. In a prospective cohort, the prevalence was reported to be 24% in patients with a tricuspid and 52% in patients with a BAV (26). According to one analysis, a dilated ascending aorta was not associated with worse procedural success rates or adverse clinical outcomes in TAVR recipients after a median follow-up of 14 months (27). Notably, in high-risk patients, the diameter of the ascending aorta remained relatively stable after TAVR. Thus, a conservative approach toward aortic dilatation in high-risk TAVR patients seems to be justified. It should be noted, however, that sufficient data in TAVR candidates with an ascending aorta of >50 mm and additional risk factors such as a BAV are not available. In intermediate- and low-risk TAVR patients, the risk of surgical aortic repair must be weighed against the patient's life-expectancy and the probability of a relevant progression of aortic dilatation. As a consequence, the optimal management in such cases remains to be determined by the heart team. While relevant aortic dilatation (>50 mm) clearly favors SAVR over TAVR, TAVR is technically feasible in many cases. In contrast, complex aortic root pathology is still considered a relative contraindication to TAVR and should be limited to highly selected patients. In this context, a transapical approach combined with a right subclavian arteriotomy as a backup arterial cannulation site in case of conversion to open heart surgery has been proposed (28).

## Challenges in Bicuspid Aortic Valve

BAV is a significant risk factor for premature aortic valve disease (29). It represents the most common cause of isolated AS in patients aged 50–70 years and is present in up to 20% of the AS in Western population over 80 years of age (30–32). The use of three-dimensional imaging, mainly ECG-gated CT, is essential in the diagnosis of BAV, as echocardiography detects congenital BAV morphology only in 58–66% of cases (33–35).

### Bicuspid Anatomy

BAV develops due to abnormal valvulogenesis. Failure of adjacent cusps to separate from each other results in the development of an aortic valve with only two cusps, with one cusp usually larger than the other. Morphology of the BAV varies according to which commissures are fused (31). At the site of failed cusps separation, there is usually a raphe, which is a prominent ridge on the middle of the outflow surface of one of the cusps extending to the aortic wall (36). Sievers and Schmidtke classified BAV according to the number of raphes into (a) Type 0 (valve with no raphe), (b) type 1 (valve with one raphe), and (c) type 2 (valve with two raphes) (37). After birth, the BAV undergoes a degenerative process with advancing age including fibrosis, calcification and myxomatous degeneration of the valve cusps. Nodular calcific and fibrotic degeneration tend to be more pronounced at the raphe. Moreover, BAV may be associated with aortic dilatation (38). A cluster analysis showed four patterns of aortic dilatation: (a) cluster I, aortic root alone, (b) cluster II, tubular ascending aorta alone, (c) cluster III, tubular portion and transverse arch, and (d) cluster IV, aortic root and tubular portion with tapering across the transverse arch (39). The clinical consequences of these changes comprise AS or aortic regurgitation (AR), endocarditis, aortic aneurysm formation, and aortic dissection (31, 40, 41).

### Procedural Considerations

In the context of BAV, SAVR remains the treatment of choice and TAVR can be an alternative to surgery in patients who are at high surgical risk (42–44). However, as lower surgical risk trials have recently shown that TAVR is non-inferior or even superior to SAVR in short term outcomes for patients with low surgical risk, the proportion of potential TAVR candidates with BAV is likely to increase in the future (6, 7, 45, 46). TAVR in a BAV has many potential challenges (40, 47–49). Balloon valvuloplasty may lead to disruption of the fused commissures, resulting in severe AR. In comparison to TAVR in a stenotic tricuspid aortic valve, the point of highest ellipticity in the stenotic BAV could be positioned above the aortic annulus, at the level of the commissures and leaflets (50), with large annular dimensions which may impair valve precise location, full apposition and sealing during TAVR resulting in a relatively greater degree of paravalvular leak (PVL). The more calcified, bulky, and asymmetrical leaflets may interfere with valve expansion and valve hemodynamics with higher transvalvular gradients and PVL. The calcified raphe may place differential stress on the expansion of the valve, increasing the risk of PVL, new pacemaker implantation, new-onset left bundle branch block, and annular rupture. Moreover, the presence of aortic disease increases the risk of dissection or rupture during valvuloplasty, post-dilatation, or implantation of balloon-expandable valves. Finally, the underexpansion and/or the non-circular shape of the THV may affect long-term durability. All these factors have an impact on short and long-term prognosis of patients with BAV undergoing TAVR.

The most appropriate sizing method for TAVR in BAV is controversial and debatable. Sizing in BAV includes 2 different methods using multidetector computed tomography; an annular sizing and a supra-annular sizing. In the supra-annular sizing, it has been suggested to measure the inter-commissural distance about 4 mm above the annular plane due to the different aortic root shapes in BAV (cylindrical, trapezoid, inverse trapezoid). In the Bicuspid Aortic Valve Anatomy and Relationship with Devices (BAVARD) registry, annulus-based sizing in BAV was valid in selecting the TAVR device size with recommending undersizing when the intercommissural distance is smaller than the mean annular diameter (inverse trapezoid anatomy) (50). Moreover, in a retrospective study comparing annular and supra-annular sizing in BAV, annular sizing was deemed to be appropriate in 96.3%, oversized in 0.5%, and undersized in 3.5% of cases; while supra-annular sizing would have resulted in a selection of similar sizes in 61.3%, upsizing in 19.8%, and downsizing in 17.5%. In this study, compared to annular sizing, supra-annular sizing may have resulted in a potential worsening of results in 36.4% of patients due to inappropriate valve size selection and a more appropriate valve size selection in only 2.3% of cases (51).

### Outcomes of TAVR in BAV

**Figure 4:**
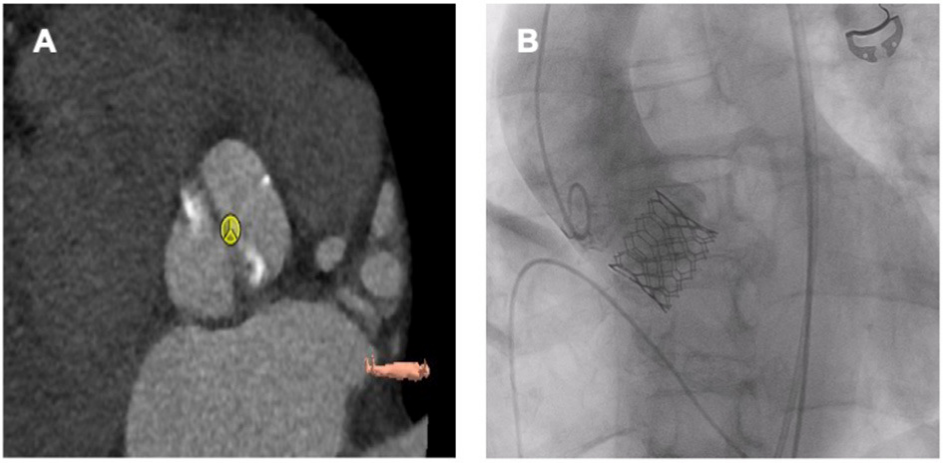
Sapien 3 Ultra 26 mm in a bicuspid aortic valve (Sievers Typ 1a R-L). **(A)** Preprocedural planning. **(B)** Fluoroscopy after TAVR.

Patients with a degenerative BAV have been excluded from major randomized and observational studies (52). This may be explained by the higher rates of PVL, need for pacemaker implantation, valve malposition, risk of annular rupture and all-cause mortality seen in the first experience with TAVR in BAV, as compared with tricuspid aortic valve (42–44, 53–56). However, these results were mostly based on small series and implantation of first-generation THVs with a limited evaluation of preprocedural computed tomography. Some of these challenges have been overcome by using new generation devices, which may offer advantages over earlier valves specially in patients with BAV. For example, the Evolut PRO valve, which is the latest member of the CoreValve family, is characterized by an external pericardial wrap to further reduce the risk for significant PVL. This external pericardial wrap increases sealing between the THV and the native anatomy (57). The Edwards SAPIEN 3 valve (Edwards Lifesciences, Irvine, California) incorporates an outer fabric seal designed to prevent paravalvular AR (58, 59). This external seal may adapt better to the irregular annuli shapes and the asymmetrically calcified leaflets in patients with BAV, thus reducing paravalvular AR in this patient group (60) (Figure 4). Studies included in the comparison of TAVR between BAV vs. tricuspid aortic valve patients using new generation devices are shown in Table 1. In a registry-based study of propensity-matched patients who had undergone TAVR for AS, patients who had BAV stenosis, compared with tricuspid AS, had no significant difference in 30-day or 1-year mortality. The stroke rate was higher in patients with BAV stenosis at 30 days but did not significantly differ at 1 year between the 2 groups. There were no significant differences in valve hemodynamics (aortic valve gradients and areas) and paravalvular AR between the 2 groups at 30 days and 1 year. Both groups had significant and comparable improvement in functional and health status after TAVR (62). In the ACURATE neo implantation in bicuspid aortic valve registry, compared to TAV, the rates of moderate perivalvular leak and stoke were significantly higher in the BAV group at 30-day follow-up; however, these differences between the two groups became non-significant after propensity score matching. Only BAV more frequently required predilation and post-dilation to achieve a satisfactory result after propensity score matching. Otherwise, the rates of pacemaker implantation, all-cause mortality, re-hospitalization for cardiovascular reasons, vascular complications, and major bleedings were similar between the two populations at 30-day follow-up (63). In a recently published large meta-analysis on TAVR in BAV patients, 30-day and 1-year mortality, the rates of stroke, vascular complications, acute kidney injury and new pacemaker implantation after TAVR did not differ between BAV and tricuspid AS patients (67). In this meta-analysis, subjects with BAV had significantly higher risk of device failure, conversion to conventional surgery, need for implantation of a second valve and moderate/severe PVL. Despite the rate of adverse events significantly decreased with new-generation devices, TAVR showed better procedural results in tricuspid AS compared to BAV, with the exception of device failure, which was similar when patients were treated with new-generation THVs (67). In a recently published study evaluating the association of BAV morphology and outcomes of TAVR with the new generation devices, calcified raphe, and excess leaflet calcification were independently associated with increased 2-year all-cause mortality. Moreover, Patients with combined calcified raphe and excessive leaflet calcium were the highest risk phenotype associated with more frequent procedural complications, such as aortic root injury and paravalvular regurgitation, and a 3-fold higher mortality (68).

**Table 1:** Studies included in the comparison of TAVR between BAV vs. TAV patients using new generation devices.

**Publication**	**Valve implanted**	**No. of patients**	**Follow-up**	**Significant differences**	**Non-significant differences**
Forrest et al. (61)	Evolut R Evolut PRO	929 in each group—PSM	Procedural, 30-day and 1-year	More patients in the BAV group required aortic valve reintervention at 30-day and 1-year	In-hospital events (mortality, stroke, coronary obstruction, pacemaker implantations, vascular complication, or post-procedural length of stay) 30-day and 1-year (all-cause mortality, stroke, pacemaker implantation, coronary intervention, or life-threatening bleeding)
Makkar et al. (62)	Sapien 3	2,691 in each group—PSM	30-day and 1-year	Stroke rate was higher in patients with BAV stenosis at 30 days	Stroke rate at 1 year Mortality Valve hemodynamics (aortic valve gradients and areas) PVL
Mangieri et al. (63)	ACURATE neo	54 in each group—PSM	30-day	BAV more frequently required pre-dilation and post-dilation	PVL Stroke Pacemaker implantation All-cause mortality Re-hospitalization for cardiovascular reasons Vascular complications Major bleedings
Yoon et al. (44)	Sapien 3 Lotus Evolut R	226 in BAV group 225 in TAV group	Procedural, 30-day and 1-year		Procedural complications (conversion to surgery, 2nd valve implantation, PVL, absence of device success, new PPM) 30-day outcomes (all-cause mortality, all stroke, life-threatening bleeding, major vascular complications, AKI) 1-year all-cause mortality
Tchetche et al. (50)	Sapien 3 Lotus Evolut R Acurate Neo Portico Direct flow	101 in BAV group 88 in TAV group	30-day	Smaller indexed orifice area in BAV patients	All-cause mortality, myocardial infarction, disabling stroke, bleeding, vascular complications, pacemaker implantation PVL, PPM
Arai et al. (64)	Sapien 3	10 in BAV group 143 in TAV group	30-day		PVL 30-day mortality Procedural success, major stroke, AKI, major vascular complications, life-threatening bleeding, annulus rupture, pacemaker implantation, need of 2nd valve
Kawamori et al. (65)	Sapien 3	41 in BAV group 239 in TAV group	Procedural and 30-day		Procedural death, Prosthesis embolization, Tamponade, Device success 30-day outcome: death, stroke or TIA, major vascular complication, bleeding (life-threatening or major bleeding), AKI, new pacemaker, early safety
Sannino et al. (66)	Sapien 3 Lotus Evolut R	22 in BAV group 182 in TAV group	1-year		1-year survival

*AKI, acute kidney injury; BAV, bicuspid aortic valve; PPM, patient prosthesis mismatch; PSM, propensity score matching; PVL, paravalvular aortic leakage; TAV, tricuspid aortic valve; TIA, transient ischemic attacks*.

## Challenges in Pure Aortic Regurgitation

While TAVR has emerged as the standard of care for patients with severe AS, the efficacy of TAVR in native pure AR (NPAR) has been largely limited by inherent anatomical differences, including the lack of a calcified native valve apparatus to anchor the THV in place, large aortic annuli, and dilation of the left ventricle (4–6).

### General Considerations

To date, there is only limited data evaluating the outcomes of TAVR in NPAR and guidelines recommend SAVR as the treatment of choice for patients with NPAR in combination with clinical symptoms, increased left ventricular dimensions, or reduction of left ventricular function (8, 9). However, advanced age and multiple comorbidities were frequent reasons for conservative rather than surgical management, resulting in an annual mortality rate of up to 20% (69). In selected patients with NPAR and high surgical risk, off-label TAVR is technically feasible with acceptable early morbidity and mortality. Lack of a native valve calcification to anchor the THV in place and the dilated aortic valve annulus represent the main challenges during TAVR in patients with NPAR. Moreover, AR is usually associated with an aortopathy characterized by dilatation of the ascending aorta. These factors make device positioning and stabilization during deployment very difficult with the potential risk of device dislodgement, embolization or malposition and subsequent moderate or severe degree of AR (70, 71). To overcome these technical challenges, valve oversizing by 15–20% has been recommended to reduce the risk of valve migration. On the other hand, oversizing should not exceed 20% due to the risk of annular rupture and conduction system abnormalities (72, 73). New-generation dedicated valve designs depend on anchoring in the aortic annulus and clipping the native valve leaflets may offer more stability during valve deployment reducing the risk of device embolization or malposition (74). In patients with a calcified aortic annulus, the radiopaque calcification at the level of the annulus acts as a fluoroscopic landmark for the operator to position the valve during deployment. Absence of radiopaque calcium in patients with NPAR increases the risk of malposition during deployment. To guide valve deployment in this case, it is recommended to place two pigtail catheters in different sinuses of Valsalva to improve visualization and provide a clearly defined fluoroscopic coplanar annular view (75). Rapid pacing during valve deployment with 180 beats per minute for balloon-expandable and 120 beats per minute for self-expanding valves may allow stable anchoring of the valve while avoiding pop-out movement during deployment.

### Non-dedicated TAVR Devices for NPAR

**Figure 5:**
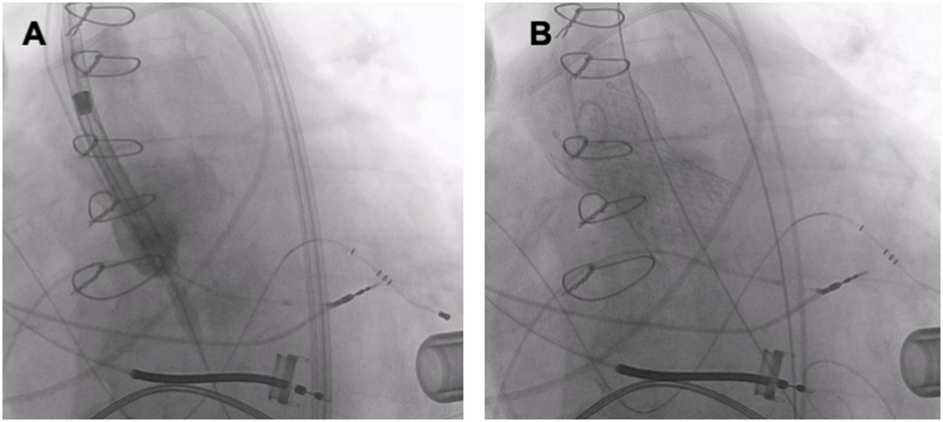
TAVR (Medtronic Evolut R 34 mm) in a patient with pure AR and a left ventricular assist device. **(A)** THV deployment. **(B)** Fluoroscopy after TAVR.

Non-dedicated THVs were originally developed to be implanted in patients with AS, as fixation of these devices depends on the calcified aortic annulus and (Figure 5). Second-generation, non-dedicated TAVR devices for NPAR can be divided into self-expanding devices (Evolut R and ACURATE neo) and balloon-expandable devices (SAPIEN 3). Compared to first-generation TAVR devices, second-generation THVs were associated with significantly lower incidences of second valve implantation and residual AR. Accordingly they achieve significantly higher procedural success rates (71, 76, 77). This may be related to availability of larger valves and the use of paravalvular skirts to prevent PVL in the second-generation devices in addition to the modified oversizing strategies. However, the rates of adverse outcome are still significantly higher than in patients with AS and the increased risk of device embolization and the frequent need for a second device must be weighted carefully against the option of conventional surgery, when considering the use of non-dedicate TAVR devices. In patients with NPAR, self-expanding THVs have been preferentially chosen to be implanted in spite of lack of valve calcification. Its self-expanding properties offer stability during device positioning, and ensure anchoring of the prosthesis even in a non-calcified annulus (74). The recapture and repositioning features after partial deployment of self-expanding THVs make the prosthesis behave in a more predictable manner (78, 79). Moreover, the Evolut PRO device incorporates an outer skirt at the lower part (ventricular side) of the stent which allows for better adhesion of the valve at its ventricular and annular level, thereby minimizing the PVL (80). The ACURATE neo valve is another self-expanding supra-annular valve. The device is implanted in a top-down two-step deployment mechanism, ensuring the release of the lower crown of the stent only when the upper crown is in proper position, with stabilization arches and an upper crown anchoring the device in the aortic annulus. This facilitates stable deployment and reduces the risk of device migration and coronary obstruction. In the absence of calcification, the protruding upper crown may serve as a safety anchor preventing its dislocation into the left ventricular outflow tract. Moreover, the stable top-down two-step deployment system may be less affected by the increased stroke volume and facilitates the accurate positioning of the THV. Furthermore, there is a skirt at the level of the annulus to minimize PVL (81). In patients with AS, transfemoral TAVR with the self-expanding ACURATE neo did not meet non-inferiority compared to the balloon-expandable SAPIEN 3 device and to the self-expanding CoreValve Evolut in terms of early safety and clinical efficacy outcomes (82, 83). However, two small multicenter experiences of the ACURATE neo THV for NPAR showed that transfemoral TAVR with the ACURATE neo THV may be an option for the treatment of pure AR in selected inoperable patients with suitable anatomy (84, 85). The Edwards SAPIEN 3 is a balloon-expandable intra-annular TAVR device. The valve is not recapturable, as it is a balloon-expandable device and hence repositioning in case of malposition is not possible. The SAPIEN 3 device incorporates a polyethylene terephthalate (PET) skirt around its outer frame. This skirt is divided into pockets and serves to capture retrograde blood that clots sealing the gap between the valve and tissue and minimizing PVL (86). Annulus oversizing of >15% using from 2 to 4 and sometimes up to 10 ml extra volume according to the left ventricular outflow tract dimensions may help to fix the device in its place (87, 88).

### Dedicated TAVR Devices for NPAR

JenaValve (JenaValve) and the J. Valve (JC Medical) are second-generation TAVR devices that rely on clip-based fixation over the native aortic valve leaflet independent of annular calcification. The JenaValve was the first dedicated device to get the CE mark, while the J valve was certified by China Food and Drug Administration for use in NPAR (89–91). The use of these “on-label” TAVR devices was associated with higher procedural success compared to other second-generation valves (76). The JenaValve is a self–expanding transapical valve with three integrated locators allowing anatomically correct orientation of the prosthesis, and a special clipping mechanism fixing the device onto the native leaflets to stabilize implantation even in the absence of annular or leaflet calcifications (92). The transapical system is no longer available since June 2016. Another NPAR-dedicated second-generation TAVR device is the J-Valve transcatheter aortic valve. The J-Valve has three U-shaped “anchor rings” and is deployed in a two-step process. First, the anchor rings are opened above the native valve and are retracted in the transapical approach or advanced in the TF approach into the valve apparatus allowing automatic anatomic alignment in the aortic sinuses and clasping of the native valve leaflets. Once positioned, the self-expanding valve is then deployed within the anchor rings and secures the native valve leaflets. The first-in-human transapical implantation was reported in 2015 and the TF implantation in 2019 (93, 94). In a meta-analysis comparing second-generation devices to J valve and JenaValve, the use of J valve or JenaValve was associated with a significantly higher procedural success without any effect on mortality or rates of > trace residual AR (77).

## Challenges in Valve-in-Valve Procedures

**Figure 6:**
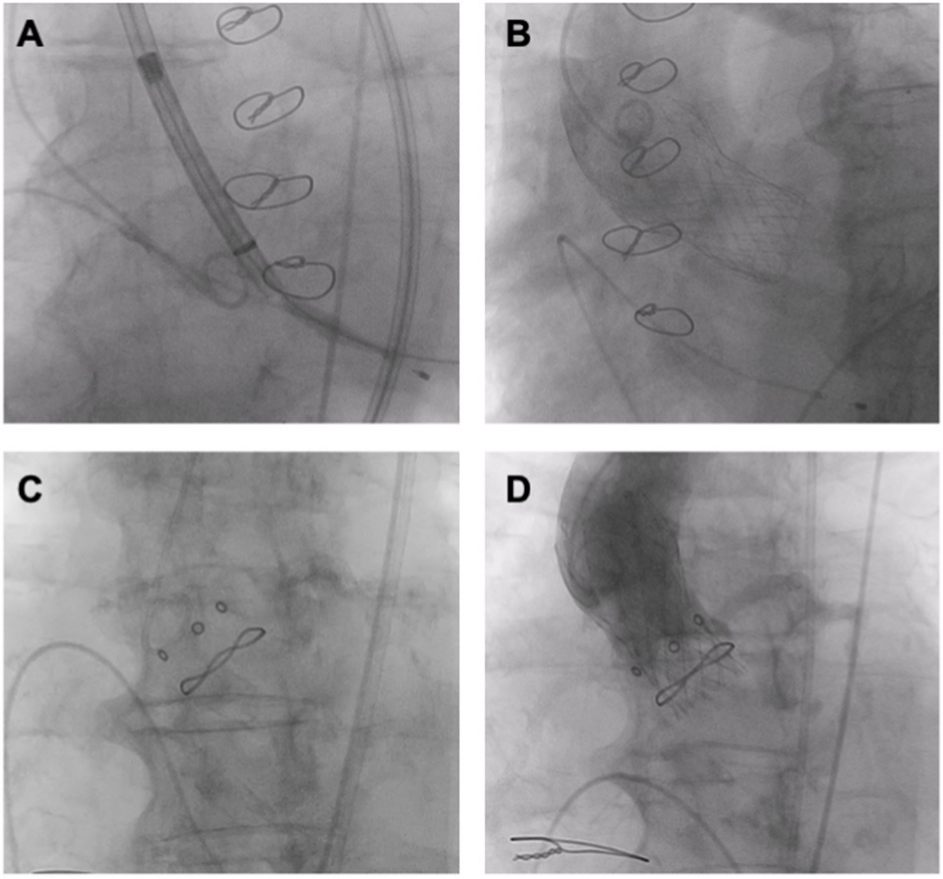
ViV TAVR. **(A,B)** ViV (Medtronic Evolut R 29 mm in Trifecta 25 mm). **(C,D)** ViV (Medtronic Evolut R 23 mm in Hancock II 21 mm).

As data on TAVR are still very limited for patients <75 years of age, current guidelines recommend SAVR in young patients with AS and low surgical risk. During SAVR, the stenotic aortic valve can be replaced with either a bioprosthetic or mechanical valve with a greater preference for bioprosthetic valves in the last two decades in comparison to mechanical prostheses (95). Accordingly, it is expected that the number of either redo-SAVR or ViV TAVR for a failed bioprosthetic valve will increase, as younger patients will require valve re-intervention earlier and more frequently compared to older patients (96). Being less invasive than conventional surgery, ViV TAVR has emerged as an attractive alternative to redo-SAVR in patients with failing degenerated bioprosthetic surgical heart valves (SHV) and guidelines recommend it as an option in patients with increased surgical risk (8, 9) (Figure 6).

### Outcomes of ViV TAVR

Current available data on ViV TAVR are obtained from retrospective studies, which showed that ViV TAVR is a feasible and safe therapeutic option with favorable acute procedural results and low complication rate. In the Valve-in-Valve International Data (VIVID) registry including 459 patients with degenerated bioprosthetic valves undergoing ViV TAVR, overall 1-year survival was 83.2% with lower survival among patients with small bioprostheses and those with predominant surgical valve stenosis (97). In the PARTNER (Placement of Aortic Transcatheter Valves) 2 VIV trial, TAVR for bioprosthetic aortic valve failure was associated with relatively low mortality and complication rates, improved hemodynamics, and excellent functional and quality-of-life outcomes at 1 and 3 years (98, 99). Interestingly, when compared to standard TAVR procedures for native AS treatment, ViV TAVR had comparable or lower 30-day mortality, lower 1-year mortality, a lower rate of moderate/severe PVL, and less frequent events of hospitalization for heart failure (100, 101). However, optimal valve and patient selection are important to avoid complications such as coronary obstruction, malposition, and residual high gradients. To guide the selection, sizing and positioning of the THV, a VIV smartphone application is widely used and provides a clear understanding of commonly used surgical valve and ring designs and their compatibility with THVs currently available with descriptions, images, dimensions, photographic and fluoroscopic images (102). The most commonly used THV devices in ViV TAVR were the self-expanding Medtronic CoreValve or Evolut R valves and the balloon-expandable Edwards SAPIEN XT or SAPIEN 3 valves. The Edwards SAPIEN XT has been approved by the FDA for ViV TAVR procedures in patients who are at intermediate- or high-risk for SAVR, while the Medtronic CoreValve obtained the FDA approval for high-risk patients for SAVR.

### ViV TAVR vs. Redo SAVR and THV in THV

Recently, many retrospective studies and meta-analyses were published comparing VIV TAVR to redo SAVR. In a propensity score-matched analysis containing 717 matched pairs, VIV TAVR was associated with lower rates of 30-day all-cause death, cardiovascular death, and new onset of atrial fibrillation compared to redo SAVR, while permanent pacemaker implantation was less often reported after redo SAVR. However, in a median follow-up of 516 days, no significant difference was observed between the 2 groups in the composite endpoint of cardiovascular death, all-cause stroke, myocardial infarction, and rehospitalization for heart failure (103). This advantage of VIV TAVR over redo SAVR regarding short term outcome was again confirmed in a large analysis of 2,181 matched pairs comparing VIV-TAVR and redo SAVR. This study has shown that VIV TAVR patients had significantly shorter hospital stay and lower 30-day mortality, 30-day morbidity and rates of major bleeding, compared to redo SAVR. These findings remained robust in the multivariate analysis, propensity-score matched analysis and in a sensitivity analysis (104).

In a recently published meta-analysis including 16,207 patients of ViV TAVR and redo SAVR, ViV TAVR was associated with lower rates of 30-day mortality, stroke, permanent pacemaker implantation, and major bleeding, as well as with shorter hospital stay. In contrast, redo SAVR was associated with lower rates of myocardial infarction and severe post-procedural patient-prosthesis mismatch. Additionally, a trend toward a lower risk for 1-year mortality was seen (105). These results were more or less consistent with another recent meta-analysis including 8,430 patients. In this meta-analysis, ViV TAVR was associated with lower procedural mortality, 30-day mortality, stroke, and major bleeding when compared with redo SAVR. Again, the mean transvalvular pressure gradient was significantly higher post-implantation in the ViV-TAVR group when compared with the Redo-SAVR arm. In a follow-up period of 1.74 years, ViV-TAVR was associated with a similar risk of all-cause mortality, cardiovascular mortality, myocardial infarction, permanent pacemaker implantation, and the rate of moderate to severe PVL, compared to redo SAVR (106).

Interestingly, THV in THV showed more favorable results compared to THV in SAV. The recently published propensity score-matched study comparing the performance of TAVR in failed THV vs. surgical bioprostheses, THV in THV was associated with more frequent procedural success compared with THV-in-SAV, driven largely by lower residual aortic valve gradient, with no difference in early safety or in mortality up to 1 year after THV in THV (107).

### Complications Related to ViV TAVR

#### Valve Embolization

Valve embolization is a serious complication during a VIV TAVR due to malposition of the THV. To avoid malposition, the THV must be optimally placed as an excessively high implantation as well as undersizing of the THV increase the risk of device malposition and consequently valve embolization (108). Stentless surgical valves have been linked to the highest incidence of THV malposition (109). However, the risk of malposition has notably decreased with increased experience, availability of repositionable and retrievable devices with a wider range of THV sizes and a thorough approach for sizing and positioning.

#### Coronary Obstruction in ViV Procedures

The incidence of coronary obstruction during ViV TAVR has been reported to be 3.5%, which is nearly 6-fold higher compared to TAVR in a native aortic valve (109). Coronary obstruction is a life-threatening complication, which is influenced by interaction between the patient's native anatomy and type of SHV previously implanted which determine the final position of the bioprosthetic leaflets that will be dislodged by the THV in relation to the coronary ostia (110). During ViV TAVR, coronary occlusion may occur due to direct contact of a bioprosthetic leaflet with the coronary ostia as a consequence of the THV expansion. Another mechanism may be accused when the bioprosthetic valve extends above the sinotubular junction (STJ) and contacts the aortic wall above a coronary ostium creating a covered cylinder in the aortic root. To predict and prevent this life-threatening complication, a thorough assessment is required before performing a VIV procedure (110). Risk of coronary obstruction is increased if the aortic root is narrow, especially with a virtual THV to coronary distance (VTC, which is the distance between the stent frame and sinus of Valsalva in cross-section of cardiac CT) of <4 mm. As SHVs are usually implanted in a supra-annular position, these patients often present with lower coronary heights. During ViV TAVR, it is important to measure the height of the coronary origin in relation to the sewing ring or the basal plane of the SHV. Stentless SHV and externally mounted leaflets of the SHV have a higher incidence of coronary obstruction as they will be implanted in a supra-annular position, their leaflets are longer, and the leaflets tend to extend outward beyond the frame of the device once the THV is expanded. Other predicting factors for coronary obstruction include SHV with pericardial leaflets rather than porcine leaflets, if bioprosthetic valve fracture is planned, and in case of absent coronary filling on BAV angiography (111, 112). An algorithm to determine the risk of coronary obstruction based on the type of the SHV, height of the coronary origin, VTC and width of sinuses of Valsalva (SOV) has been suggested for planning of ViV TAVR and further management. This algorithm suggests that low-risk patients for coronary obstruction should undergo VIV without further action. These low-risk patients are patients with: (a) high coronary origins (take-off above the posts of stented SHVs or >12 mm from the valvular annulus of a stentless SHV), (b) a stented bioprosthesis and VTC >4 mm, especially with internally mounted leaflets valves, and (c) stentless valves, but wide SOV and high STJ. On the other hand, in those with high-risk features (low coronary ostium and VTC <4 mm in stented SHVs or shallow SOV in stentless SHVs), patients should be discussed individually in heart-team and should be considered either for redo surgery, VIV with an upfront coronary protection strategy, or keeping the patient on medical treatment (113). Upfront coronary protection strategies include mainly chimney stenting technique and BASILICA (Bioprosthetic or native aortic scallop intentional laceration to prevent iatrogenic coronary artery obstruction during TAVR) technique. The chimney technique offers a potential predictable stepwise method of coronary protection in which a coronary guidewire, preferably a guide-extension catheter, and an undeployed long coronary stent are positioned in the peripheral part of the left anterior descending artery. After implantation of the THV, the patency of the coronary artery is assessed by direct contrast injection through the guide-catheter or through an aortography. In case of coronary obstruction, the undeployed stent is pulled back and deployed at the coronary ostium with some protrusion into the aorta, with the proximal parts placed between the aortic wall and the bioprosthetic leaflets, in order to push the SHV leaflets away from the ostium and to maintain coronary flow (114). Long-term outcome data of this technique are not available and long-term patency of this strategy, is unclear. Mechanical deformation of the stent by the TAVR prosthesis is possible, retrieving undeployed stents is sometimes not easy and future access to the coronary arteries would be very difficult (115, 116). The second coronary protective strategy is the BASILICA procedure, which has recently emerged as a method for disrupting bioprosthetic leaflets bevor THV implantation in patients undergoing ViV procedures and at high risk of coronary occlusion (117). In the BASILICA procedure, a multipurpose guiding catheter with a combination of guidewire and microcatheter is advanced to the coronary cusp targeted for laceration (usually the left coronary artery). After penetration of the cusp with an electrified wire, the wire is snared in the left ventricular outflow tract and externalized. The coronary cusp is lacerated in their midline with catheter electrosurgery, allowing them to splay laterally as they are displaced outwards by the THV, thereby creating a triangular space that allows blood flow into the coronary artery. The BASILICA trial prospectively enrolled 30 patients at high risk of coronary artery obstruction and reported 100% freedom from coronary obstruction and 93% procedural success (118).

Another strategy is to use partially retrievable THVs, such as the Evolut-R and Portico valves, and to assess coronary flow status before complete deployment of the THV. In case of coronary occlusion, the THV can be retrieved with planning an upfront coronary protection technique, discuss the case for redo surgery or keeping the patient on maximal medical therapy. However, if coronary obstruction occurs after deployment of the THV, the first best option is to try emergent PCI and provide hemodynamic support in case of circulatory deterioration. If this is not possible, urgent coronary artery bypass graft surgery may be required (112). If coronary revascularization cannot be achieved and the hemodynamics are poor, the THV can be snared (Medtronic CoreValve), or removed from its anatomical position by using an oversized balloon and pulled up out into the ascending aorta to maintain coronary flow (119). Delayed coronary obstruction is a rare complication that occurs more frequently after VIV procedures and with self-expanding devices than balloon-expandable valves. Delayed coronary obstruction may be related to progressive expansion of self-expanding THVs within hours/days after the procedure causing obstruction by the surgical prosthetic aortic leaflets. Mechanisms of late coronary obstruction (>7 days) include endothelialization of surgical bioprosthetic leaflets, or embolization of thrombus that may occur in the THV or SOV (120).

#### High Residual Gradients

ViV TAVR may be a strong predictor for residual high gradients which was associated with increased risk of mortality at 1 year (97, 121). Residual high gradients are usually observed in SHVs with a true inner diameter of <21 mm (109). Compared to balloon-expandable valves, self-expanding THVs are associated with lower gradients after ViV TAVR, especially in the presence of preexisting severe patient-prosthesis mismatch with a gradient ≥20 mmHg (109). *In-vitro* studies have shown that higher deployment of the THV in ViV TAVR resulted in lower gradients and greater effective orifice areas, as leaflets in the supra-annular position are better able to expand and coapt without the constraint of the fixed basal surgical valve ring (122, 123). Bioprosthetic valve fracture (BVF) has emerged as an alternative to redo surgery or ViV TAVR in small bioprostheses with residual high gradients (124). This technique essentially involves fracturing of the inflow ring of an SHV by means of a high-pressure, non-compliant balloon placed across the valve ring during rapid ventricular pacing. This increases the inflow diameter by at least one size and allows optimal expansion of the THV device and thus reduces gradients. Only the Abbott Trifecta and Medtronic Hancock II valves are not fracturable (125). Concerns about the risks of annular rupture (especially in heavily calcified annulus and LVOT) and damage to the THV leaflets with the high-pressure balloon warrant further evaluation.

#### Valve Thrombosis

Reduced leaflet motion caused by thrombosis and leaflet immobility have been seen after TAVR. ViV TAVR has been identified as a risk factor for leaflet thrombosis, a complication that can affect THV durability and lead to premature structural valve deterioration or stroke (126–129). Leaflet thrombosis is often subclinical, which can be detected in echocardiography through elevated transaortic gradients. However, “4D” volume-rendered CT is the gold standard for diagnosis. Anticoagulated patients appear less likely to develop leaflet thrombosis and there have been case reports of early or delayed leaflet thickening or thrombosis responding to anticoagulation, leading some to recommend a period of anticoagulation after VIV procedures (130).

## Challenges in Coronary Anatomy

The rate of coronary artery disease (CAD) among patients referred for TAVR varies between 40 and 75% (131). While CAD is significantly less prevalent among low-risk patients, challenges in coronary anatomy remain an important issue in the context of TAVR (6, 7). These include (a) low coronary ostia, (b) optimal revascularization strategy, and (c) coronary access after TAVR.

### Low Coronary Ostia

A short distance between the aortic annulus and the coronary ostia may pose a considerable challenge due to the potential risk of coronary obstruction. Therefore, in the presence of low coronary ostia, guidelines support the use of SAVR in preference to TAVR (8, 9). While there is no clear cut-off for a minimal coronary ostium height, a distance of >10 mm is generally regarded as safe in most patients with native AS. Conversely, patients with a coronary ostium height of ≤10 mm have been reported to be at elevated risk of coronary obstruction (132, 133). Coronary obstruction is a rare complication of TAVR with a reported incidence of <1% among TAVR patients with native AS and an incidence of ~2% in patients undergoing ViV procedures (112, 134). Coronary obstruction predominantly affects the left coronary ostium, usually leads to immediate hemodynamic deterioration and is associated with a 30-day mortality rate of up to 35% (133). In native aortic valve disease, it is mostly caused by displaced calcium from the aortic valve cusps; in patients undergoing ViV TAVR, calcified prosthesis components and prosthetic leaflet avulsion are important factors (110, 111). Furthermore, a narrow aortic root (<28 mm at the SOV), a large native aortic valve leaflet and a BAV have been identified as additional anatomical risk factors (132, 133). While coronary obstruction is typically encountered during the procedure, cases of delayed coronary obstruction (>7 days) have been reported (120). There are no randomized clinical trials evaluating the optimal valve type in the context of low coronary ostia. Based on a multicenter registry, balloon-expandable valves may be associated with a significantly higher risk of coronary obstruction compared to self-expanding valves (111). However, coronary protection as well as future coronary accesses may be more challenging in patients receiving a self-expanding valve. In addition, the risk of delayed coronary obstruction seems to be higher than in patients treated with a balloon-expandable valve (120). Therefore, the choice of a specific valve should be based on anatomical, clinical and procedural aspects as well the expertise of the local heart team. In case of unexpected coronary obstruction, emergency percutaneous coronary intervention is the preferred treatment of choice with success rates of >80% (111). However, it may be necessary to deploy a second stent in order to achieve adequate stent expansion against the compression by the THV (132, 135). In addition, emergency coronary artery bypass grafting in the hybrid OR may be necessary in a subgroup of patients. In patients who are at elevated risk of coronary obstruction protective measures should be taken. In the majority of cases pre-emptive wiring of the coronary ostia is performed before valve deployment. In high-risk patients, this involves pre-emptive stenting of the coronary ostia using the chimney technique, as this strategy has been associated with improved outcomes compared to pre-emptive wiring alone (114, 136). In addition, the aforementioned BASILICA procedure has emerged as a novel leaflet splitting technique in TAVR patients, especially in those undergoing ViV procedures (137).

### Optimal Revascularization Strategy

CAD is common among elderly AS patients as both entities share common risk factors (138). In the context of SAVR, concomitant myocardial revascularization is recommended (class I) in patients with significant CAD (≥70% reduction in luminal diameter) and should be considered (class IIa) in patients with moderate-severe CAD (≥50–70% stenosis) (8). In contrast, the prognostic impact of CAD and the optimal revascularization strategy among TAVR patients still remain controversial (139–141). In the absence of adequately powered randomized clinical trials, PCI is currently recommended in TAVR patients with left main or significant (≥70% stenosis) proximal CAD (8, 9). However, this approach must be individualized to the patient based on clinical (e.g., limited life-expectancy), anatomical (e.g., lesion complexity), and procedural (e.g., potentially impaired coronary access after TAVR) aspects. In the future, specific hemodynamic assessment using fractional flow reserve (FFR) or instantaneous wave-free ratio (iFR) may play an important role in the workup of CAD pre-TAVR. In addition, a combined procedure (TAVR and PCI at the same time) may be an option in low-risk TAVR patients with non-complex CAD and normal kidney function.

### Coronary Access After TAVR

Due to the high prevalence of CAD among TAVR recipients, a significant number of patients (≥10%) experience coronary events after TAVR (142). With the expansion of TAVR toward a lower-risk population, the need for coronary access after TAVR will continue to rise as underlying CAD progresses over time. Coronary access after TAVR may be challenging due to various factors including STJ diameter, coronary height, type of THV and valve implantation depth (143). Currently, there is still a paucity of sufficient data regarding feasibility, success rate, and optimal catheter selection for coronary access and PCI after TAVR. Based on clinical registries, coronary access after TAVR can be safely achieved in ≥90% of patients, but success rates may be impaired in the context of the supra-annular self-expanding CoreValve/Evolut (144–146). In fact, this observation was supported by Barbanti et al. in the RE-ACCESS (Reobtain Coronary Ostia Cannulation Beyond Transcatheter Aortic Valve Stent) Study. The authors described that unsuccessful coronary access was almost only observed in patients receiving a CoreValve Evolut R/PRO prosthesis (147). This appears to be intuitive as the CoreValve has a supra-annular fixation and a closed-cell frame design in contrast to the balloon-expandable SAPIEN valve platform. In addition, commissural malalignment seems to be an important contributor to the impairment of coronary access using the CoreValve Evolut R/PRO prostheses (148). Thus, the SAPIEN 3 seems to offer better coronary access and may be preferred over CoreValve Evolut R/PRO in patients in whom future coronary access is expected to be necessary.

In a trial to optimize commissural alignment of CoreValve Evolut R/PRO, a specific orientation of the valve catheter during implantation is recommended. Positioning the Evolut THV “Hat” marker at the outer curve of the ascending aorta during initial deployment improved commissural alignment and significantly reduced coronary artery overlap. While specific initial SAPIEN 3 orientation had no impact on alignment, having a commissural post of the ACURATE-neo THV at center back or inner curve at initial deployment improved commissural alignment and reduced coronary artery overlap (149).

Regarding the optimal catheter selection for coronary access, Yudi et al. proposed an algorithm for both self-expanding and balloon-expandable valves (143). As coronary access after TAVR remains challenging in certain scenarios, these patients should ideally be treated in specialized centers with an experience in TAVR procedures. Coronary access may be particularly challenging in patients after ViV procedures with reported failure rates of >30% (150). Modifications in valve design as well as the introduction of dedicated coronary catheters are necessary to facilitate coronary access after TAVR and ViV procedures in the future.

## Conclusion

TAVR has emerged as an established treatment option for patients with aortic valve disease across the whole spectrum of surgical risk. In light of the expansion toward a younger, lower-risk group, TAVR will be used in an expanding patient population posing a variety of concomitant anatomical and clinical challenges. While many of these difficulties can be overcome with contemporary THVs and techniques, TAVR will need to constantly evolve in the future.

## Author Contributions

MS and HS drafted the manuscript. DF critically revised the manuscript. All authors read and approved the submitted version.

## Conflict of Interest

DF is a consultant for Edwards Lifesciences and Medtronic and has received research funding from Edwards Lifesciences. The remaining authors declare that the research was conducted in the absence of any commercial or financial relationships that could be construed as a potential conflict of interest.
